# **‘**A system that is struggling’: understanding health protection resilience in England during the COVID-19 pandemic through the experiences of local health protection responders

**DOI:** 10.1186/s12913-024-10651-7

**Published:** 2024-02-08

**Authors:** Suzanne Rotheram, Stephen Clayton, Ian Buchan, Sam Ghebrehewet, Ben Barr

**Affiliations:** 1 National Institute of Health Research, Health Protection Research Unit in Gastrointestinal Infections, The University of Liverpool, Liverpool, United Kingdom; 2 Department of Public Health, Policy and Systems, The University of Liverpool, Whelan Building, Liverpool, L68 3GB United Kingdom; 3 The UK Health Security Agency (UKHSA), UK Health Security Agency North West, Cheshire and Merseyside Health Protection Team, Suite 3B, 3rd Floor, Cunard Building, Water Street, Liverpool, L3 1DS United Kingdom

**Keywords:** Qualitative research, COVID-19, Infectious diseases, Health protection, Resilience

## Abstract

**Background:**

Local health protection systems play a crucial role in infectious disease prevention and control and were critical to COVID-19 pandemic responses. Despite this vital function, few studies have explored the lived experience of health protection responders managing COVID-19. We provide new insights by examining how COVID-19 shaped infectious disease prevention and control in local health protection systems in England.

**Methods:**

Semi-structured interviews were conducted with twenty local health protection responders from three contrasting local authority areas, and Public Health England (PHE) health protection teams, in England between June 2021 - March 2022. Participants were from: PHE health protection teams (*n*=6); local authority public health teams (*n*=5); local authority Public Protection Services (*n*=7); and local authority commissioned Infection Prevention and Control Teams (*n*=2). Data were analysed using reflexive thematic analysis.

**Results:**

First, participants acknowledged the pandemic caused an unprecedented workload and disruption to local health protection service delivery. There was not enough capacity within existing local health protection systems to manage the increased workload. PHE health protection teams therefore transferred some COVID-19 related health protection tasks to other staff, mainly those employed by local authorities. Second, health protection responders highlighted how COVID-19 drew attention to the weaknesses in local health protection systems already stressed by reduced funding in the years leading up to the pandemic. Injecting money into the COVID-19 response did not completely overcome former losses in specialist health protection workforce. Third, health protection responders described how pandemic management raised the profile of public health, especially infectious disease prevention and control. Managing COVID-19 strengthened collaborative working, resulting in enhanced capacity of local health protection systems at the time.

**Conclusion:**

The COVID-19 pandemic challenged the public health preparedness of all countries. Health protection responders in this study also expressed many challenges. There was insufficient resilience in these local health protection systems and an inability to scale up the specialist health protection workforce, as required in a pandemic situation. The UK needs to learn from the pandemic experience by acknowledging and addressing the challenges faced by local health protection responders so that it can more effectively respond to future threats.

**Supplementary Information:**

The online version contains supplementary material available at 10.1186/s12913-024-10651-7.

## Introduction

To date, the SARS-CoV-2 virus has caused over 771 million cases of COVID-19 and 6.9 million deaths [1]. Pandemic management has varied greatly across the globe [2], but it has been suggested that countries with pre-existing, ‘resilient’, health systems may have had more success responding to COVID-19 [3]. Despite the more frequent application of the concept of resilience to health systems since the COVID-19 pandemic, there is still some confusion about what resilience means, how it can be assessed and what can be done to strengthen it [4]. Some definitions of resilience, such as that put forward by Thomas et al., (2020) do not require a crisis, or shock as a precondition for judging resilience within a health system [4, 5]. Instead, resilience in this context emphasises the importance of preparedness for catastrophic events within normal health system functioning, as well as how the system functions and responds to a crisis when it appears [4, 5].

In contrast, other definitions of health system resilience, while also recognising the importance of normal health system functioning, place more emphasis on preparation and response in the context of a crisis situation (e.g. a pandemic) and the development of improved performance once the crisis is over. One such definition, coined by Kruk et al., (2015) describes resilience as, *‘the capacity of health actors, institutions and populations to prepare for and effectively respond to crises; maintain core functions when a crisis hits; and, informed by lessons learned during the crisis, re-organise if lessons require it’* [6]. As we move away from the acute phase of the COVID-19 pandemic, it is important that countries reflect on their management of COVID-19, so that resilience can be built to future pandemics [3]. This is of particular consequence in the context of the United Kingdom (UK) as it was consistently ranked among countries best-prepared to withstand a pandemic, but experienced some of the worst death rates due to COVID-19 [7, 8].

An important way of gaining insights into different aspects of the resilience of health systems is to examine the lived experience of professionals working on the front line of the COVID-19 response. A large number of qualitative research studies, from a range of countries, have done this by accessing the experiences of people involved in the frontline healthcare response to the COVID-19 pandemic, i.e. healthcare staff such as doctors and nurses managing or treating patients with severe COVID-19 (e.g. [9–11]). A systematic review of qualitative studies exploring the experiences of key actors and organisations found that healthcare workers such as doctors and nurses, while experiencing extreme workloads, emphasised their resilience as they took on new responsibilities during the pandemic [11]. The authors of the study questioned this resilience, however, in light of the detrimental impact of the pandemic on the physical and mental health of participants [11].

Fewer studies, have examined the experiences of professionals involved in the public health response to the COVID-19 pandemic. By public health response, we refer to the work of public health staff such as consultants in public health, Infection Prevention and Control Nurses and Environmental Health Officers. These public health staff were not directly responsible for COVID-19 patient care but instead responsible for actions such as: providing infection, prevention and control advice; organising COVID-19 testing; providing epidemiological analyses of COVID-19 infection spread; rolling out vaccination programmes and carrying out contact tracing. The few studies that have been published in this area have included a variety of professionals such as contact tracers, border management, behavioural scientists and laboratory staff [12–18]. These studies found that public health staff, in common with healthcare staff, also experienced extreme workload increases and stress during the pandemic. Public health staff described experiencing interconnected challenges related to planning, leadership, equipment, partnerships, space, workforce, and funding - often while working within public health systems that were underfunded and understaffed [12–18]. This paper adds to this small but important body of literature, by exploring the lived experience of public health staff working in health protection roles linked to Public Health England (PHE) (UK Health Security Agency predecessor) and local authorities in England (described in more detail below) during the pandemic.

A comprehensive understanding and interrogation of the resilience of public health systems in England during the pandemic is beyond the scope of this paper. However, by making visible how the pandemic shaped the work of health protection responders, we offer some insights into what resilience in local health protection looked like in practice during this time. Before describing these findings in more detail, we start by outlining the recent history of health protection provision in England, focusing on the context of infectious disease control. This sets the scene for our findings.

## Background

**Fig. 1:**
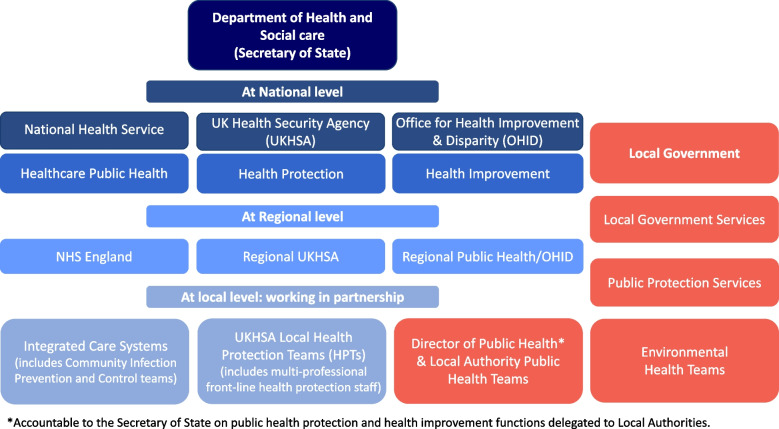
Public health System national, regional and local structures Schematic overview by the authors of infectious disease focused health protection services, what we refer to in this paper as, ‘local health protection systems.’ Note: In some areas, Community Infection Prevention and Control services/teams are commissioned or delivered by local authorities. All local government services work closely/in partnership with local public health teams

The term ‘health protection’ is a recent description of a subset of functions within public health services. Health protection functions work, through collaborative efforts, to protect individuals, groups, and populations from the impact of: infectious diseases; radiation; and chemical and environmental hazards [19]. In England, health protection is divided into four distinct areas – chemical hazards, emergency response, infectious diseases, and radiation [20]. In this paper, we focus on health protection work related to infectious disease prevention and control in local authority areas, which we refer to as ‘local health protection systems’ (Fig. 1).

### Structure of health protection provision in England 2003-2023

Between 2003 and 2013, national health protection in England was managed by the Health Protection Agency (HPA), an arms-length body that sat outside the Department of Health and the National Health Service (NHS) [19, 21]. In 2013 the HPA was abolished and amalgamated with several other ‘expert bodies’ to bring together, for the first time, public health responsibilities within one national organisation - Public Health England (PHE) [19, 22, 23]. At the same time, public health teams, led by Directors of Public Health, were moved out of the NHS and into local authorities, where Environmental Health Officers (EHOs) were also based [24, 25]. These re-organisations meant that while many health protection responsibilities lay within PHE, strategic roles around Emergency Preparedness, Resilience and Response were shared between the Directors of Public Health, PHE, the NHS and ambulance services [22, 26, 27]. A restructuring on this scale demanded local Memoranda of Understanding, as well as national guidelines to ensure clarity between these different parts of health protection as to their respective roles in the event of a major incident [22, 27, 28]. This structure was still in place at the start of the pandemic. In 2021, in the middle of the pandemic, PHE was abolished and merged with two organisations created in response to COVID-19 – Test and Trace and the Joint Biosecurity Centre – to create the UK Health Security Agency (UKHSA) [29–31] (Fig. 1).

Our study, conducted in the second half of the pandemic, therefore took place against a backdrop of two major restructures of health protection in the previous 10 years. This has led to a local health protection system structure with roles split across a national agency with regional teams (PHE/UKHSA) separate from local government departments (Public Health) and the NHS. A simplified overview of the health protection responders currently involved in local infectious disease control in England is illustrated in Fig. 1.

### Funding of health protection in England

In addition to the changes to health protection structures over the last decade described above, health protection in England has also experienced challenges in relation to UK government fiscal policies. The policy of austerity, introduced to reduce public spending following the financial crisis in 2008, has affected both public health and local authority grants. Public health grants experienced real-term funding cuts of 26% per person between 2015/2016 and 2023 [32]. Local government also experienced deep central government funding cuts of around 50% between 2010 and 2017, with EHO numbers falling nationally by 16.1% between 2009/10 and 2018/19 [33, 34]. These funding cuts have not been equally distributed – funding cuts both to local government and public health grants have been greater in areas defined as disadvantaged [32, 35]. This decade of cuts has left local authority public health, and system-wide health protection, depleted.

These dual challenges for local health protection systems – multiple re-organisations, and cuts to budgets, are important contexts in which to interpret our findings.

## Methods

This paper draws on data from a wider study which aims to understand local health protection system adaptation and resilience during periods of austerity and pandemic [36]. In this paper, we focus on how the pandemic shaped local infectious disease prevention and control in England. To do this we recruited 20 health protection responders from PHE/UKHSA and local authority health protection systems. In this paper, we use the term ‘health protection responders’ to refer to staff from across the local health protection system (Fig. 1). We cite specific roles within this system when the context requires it. All participants had some responsibility for infectious disease control in their normal role and were involved in some aspect of the COVID-19 response in England. Participants included staff working in: PHE/UKHSA teams (depending on the date of the interview) (*n*=6); local authority public health teams (LAPHT) (*n*=5); local authority Public Protection Services (PPS) (here defined as trading standards, environmental health and licensing) (*n*=7); and Community Infection Prevention and Control Teams (IPCT) (*n*=2) (Table 1).

**Table 1:** Pseudonymised participant information table

**Name (Pseudonym)**	**Area**	**Team**	**Role**	**Time in role (years)**
Joanne	LA1	Public Protection Services	EHO	20-30
Helen	LA1	Local Authority Public Health Team	Public Health Consultant	1-10
Clive	LA1	Local Authority Public Health Team	Public Health Consultant	1-10
Sarah	LA2	Public Protection Services	EHO	30-40
Michelle	LA2	Public Protection Services	Manager	20-30
Gary	LA2	Public Protection Services	Head of Service	1-10
Rachel	LA2	Local Authority Public Health Team	Public Health Consultant	1-10
Amy	LA2	PHE/UKHSA regional team	Consultant in Health Protection	1-10
Elizabeth	LA2	PHE/UKHSA regional team	Consultant in Health Protection	1-10
Ruth	LA2	PHE/UKHSA regional team	PHE (UKHSA) Scientist	10-20
Thomas	LA2	Infection Prevention and Control Team	Community IPCT lead	20-30
Louise	LA3	Public Protection Services	EHO	30-40
Andrew	LA3	Public Protection Services	Manager	10-20
Miranda	LA3	Public Protection Services	Manager	20-30
Alistair	LA3	Local Authority Public Health Team	Public Health Consultant	1-10
Tracy	LA3	Local Authority Public Health Team	Public Health Consultant	10-20
James	LA3	PHE/UKHSA regional team	Consultant in Health Protection	10-20
Sally	LA3	PHE/UKHSA regional team	Lead Health Protection practitioner	10-20
Hannah	LA3	PHE/UKHSA regional team	PHE (UKHSA) Scientist	20-30
Philippa	LA3	Infection Prevention and Control Team	Community IPCT lead	1-10

*LA* Local Authority, *EHO* Environmental Health Officer, *IPCT* Infection Prevention and Control Team, *PHE* Public Health England, *UKHSA* UK Health Security Agency

Non-PHE participants were linked to three local authorities which contrasted in terms of structure and level of deprivation. LA1 was a unitary authority in the highest quintile of deprivation; LA2 was a metropolitan borough authority in the highest quintile of deprivation; and LA3 was a district council from the lowest quintile of deprivation (See [37] for explanations of these structures). PHE/UKHSA participants were recruited if they were identified as working alongside health protection responders within these three local authorities.

Ethical approvals were received from the University of Liverpool (Reference 9927). Public and patient involvement was integrated throughout the research project (see GRIPP2 reporting, S1). Online interviews conducted by SR using Microsoft Teams took place between June 2021 and March 2022. Interviews spanned the transition of health protection responsibilities from PHE to the UKHSA and the time period between the end of the second lockdown in the UK and most COVID-19 restrictions finishing [38]. Participants gave written, informed consent to take part and were told that they could withdraw from the study at any time until the results had been published.

The semi-structured interview schedule was developed for this study (see interview schedule, S2) and focused on participant’s roles in infectious disease control and if or how budget cuts and COVID-19 had shaped their work. Interviews were recorded and transcribed verbatim. Data analysis was informed by Braun and Clarke’s reflexive thematic analysis [39]. Transcripts were first read through, and initial notes were made. Next, ‘codes’ or units of meaning were assigned to sections or lines of the data. Once codes had been assigned, these codes were grouped into ‘themes’, i.e. larger units of meaning that encompassed groups of codes which shared patterns of meaning. Coding and themes were discussed with the research team and refined throughout the process to make sure that the data within the themes were internally coherent [40].

Our three themes give insights into the enormous impact that COVID-19 had on our participants and their work around infectious disease control during the pandemic. We first show the huge disruption that COVID-19 caused. Then we demonstrate how COVID-19 drew attention to the cracks in a stressed local health protection system. We finish by showing how, in contrast to the de-prioritisation of health protection in the pre-pandemic years, COVID-19 created an opportunity for local health protection infectious disease control work to thrive.

## Results

### COVID-19 as a massively disruptive event

Our first theme shows the disruption that COVID-19 caused to the local health protection systems included in this study: First, by the workload created by managing outbreaks; second in the additional work that COVID-19 created, which we refer to as COVID+ work; and third, because COVID-19 led to infection prevention and control roles and tasks being rapidly shifted within and then beyond teams with health protection expertise.

#### Managing COVID-19 outbreaks

Without exception, all participants described the huge, overwhelming nature of their work during the COVID-19 pandemic. Workloads included many activities that would routinely be associated with the management of infectious disease outbreaks, but on a vastly expanded scale. These activities included: collating and analysing outbreak data; attending or leading COVID outbreak management meetings; disseminating infection, prevention and control advice; putting COVID-19 testing in place; rolling out community vaccination programmes; attending stakeholder meetings; supporting people as they followed self-isolation rules; and carrying out contact tracing. Many teams moved from providing a 9-5, 5-day-a-week service to a 7-day-a-week service to try and cope with the demand.

Participants often had decades of experience in health protection (Table 1), which made the language they used to describe their work during the pandemic even more significant. The work was described as *“relentless”* (Louise, LA3), *“unprecedented”* (Gary, LA2), *‘overwhelming’* (Elizabeth, LA2), *‘enormous’* (Andrew, LA3), *‘intense’* and *‘exhausting’* (Rachel, LA2) with existing resources being *‘completely swamped’* (Hannah, LA3). Thomas, who had managed outbreaks in care homes throughout the pandemic explained:

“… we were just overwhelmed, we couldn’t cope… […] we were buckling, just buckling under the pressure.” (Thomas, IPCT, LA2).

When asked what a typical working day looked like, Philippa, an Infection Prevention and Control Nurse (IPCN) described both the overwhelming numbers of outbreaks her team were dealing with, and the frustration that they experienced trying to get ahead of the Omicron SARS-CoV-2 variant(s):

“…. I have to say since December, we felt more like data analysts. We had 179 outbreaks on our COVID dashboard at one point, […] and actually the team were extremely frustrated because it didn’t matter what you threw at Omicron […] this virus was just spreading.” (Philippa, IPCT, LA3)

The sheer scale of the number of infections and outbreaks produced by successive COVID-19 waves was overwhelming to manage. This was not, however, the only type of work participants had to manage, as we describe below.

### COVID + work (COVID generated additional work)

Many participants described having to adapt their normal working practices when the UK went into lockdown [38]. Initially, these adaptations involved changing existing working practices to work remotely. For example, IPCNs managed COVID outbreaks in nursing homes over the phone, rather than their usual practice of visiting homes in person. Thomas justified this move to working remotely to protect IPCNs and ensure they could continue to manage outbreaks:

“… movement was stopped because the perception was we didn’t want to expose such a specialist finite resource because we couldn’t afford people going off sick - it would just cripple us even more.” (Thomas, IPCT, LA2)

As the pandemic progressed, health protection responders became responsible for ensuring that COVID-19 guidance issued by the UK government was interpreted correctly and followed [38]. COVID+ work therefore expanded to include: interpreting, translating, and disseminating new COVID-19 legislation as it was introduced; inspecting businesses to ensure they were following social distancing rules; and investigating reported ‘breaches’ of COVID-19 rules by businesses. Disseminating and implementing guidance created a great deal of additional work for local health protection responders, as Gary, who worked in Public Protection Services linked to LA2 explained:

“We issued about 10 versions of a letter, every time the legislation changed we would rewrite the letter, to say this is what it means.” (Gary, PPS, LA2)

As PHE was the national lead for health protection, local partners often looked to the organisation for advice. As COVID-19 guidelines were drafted centrally and changed regularly without local PHE being given advance warning of the changes, this made PHE’s advisory role particularly difficult, as Elizabeth made clear:

“[The PHE Health Protection Team] really struggled, national guidance and policy was changing on a daily basis […] everyone was coming to us for advice and we were in the same position as everybody else, so you know we were reading guidance and advice for the first time as well.” (Elizabeth, PHE/UKHSA, LA2)

The pandemic response in England therefore not only created routine outbreak work on a massively expanded scale, but also COVID generated additional work for health protection responders. The effort required to manage this COVID+ work was exacerbated by rapidly changing regulations. In order to manage all this work, the roles and tasks required to manage the pandemic had to shift significantly. The way this happened in practice is set out next.

#### Shifting roles and scaling up work

At the start of the pandemic, the responsibility for managing and responding to outbreaks of infectious diseases sat with PHE. As the number of outbreaks intensified, and the capacity within the teams that would usually manage outbreaks became insufficient, PHE initially brought in PHE staff who were not routinely involved in this work to support their response. As the number of outbreaks increased, the extra capacity created by bringing in these extra staff was not sufficient, and, as James explains, PHE was unable to cope with the demand:

“… PHE and UKHSA were unable to deliver the operational and tactical strategic response to this pandemic…” (James, PHE/UKHSA, LA3)

Local authority public health teams described how, as they became overwhelmed, PHE delegated the oversight of many COVID-19 outbreaks to local authority public health teams. Rachel, a consultant in public health, working in LA2’s public health team described how she suddenly found herself leading on work that she had training for, but which, pre-pandemic, she had limited exposure to:

“…. before transition back in 2013 PHE were very adamant that they were responsible for leading the response to outbreaks. Since the pandemic, because PHE just hasn’t had the capacity, I have never done so much health protection reactive work […]. The responsibilities have very definitely shifted in terms of what we need to do.” (Rachel, LAPHT, LA2)

This pattern of shifting roles and tasks associated with managing COVID-19 to other teams to scale up the work then played out again in local authority public health teams as they, in turn, became overwhelmed. Initially, this shifting of tasks was kept within teams linked to local authorities with health protection expertise - such as Infection Prevention and Control Teams and Environmental Health Officers (EHOs). In time, the capacity of these health protection responders was also overwhelmed. IPCNs, EHOs and local authority public health teams therefore reached out to colleagues with no, or very limited, health protection expertise, and whose usual roles were diminished by the pandemic. For example, public health teams in LA1 brought in leisure staff to help; EHOs in LA2 enlisted the help of colleagues in housing and trading standards; and Infection Prevention and Control Teams reached out to people in the education department, as Thomas described:

“…And at that point we had the schools go back […] we were supporting initially the local authority in dealing with that, but we couldn’t sustain that so that was handed over to a team in the local authority which was made up by people in the education department there and they were just given information around what they needed to advise the schools and they just ran with that.” (Thomas, IPCT, LA2)

This shifting of COVID-19 tasks and roles and scaling up of work appears to have happened in an unplanned way. It highlights a lack of capacity in health protection systems in these local authorities in England pre-pandemic, and suggests that the existing planning did not cover the scenario that unfolded during the COVID-19 pandemic.

### Drawing attention to the cracks

Our second theme describes how COVID-19 drew attention to the cracks in a local health protection system significantly weakened by funding cuts: first, by making participants confront the reduced capabilities of health protection services; second by revealing how gaps in expertise could not be rapidly ‘fixed’ by investment; and third by staff having to prioritise COVID-19 over many other health protection roles (enabled by reduction in other infectious disease incidents).

#### Confronting weakened health protection capabilities

Many participants described how, before the pandemic, health protection infrastructure and staffing had been significantly reduced. Participants described reductions in: PHE laboratories and staff; EHO staff; consultants in local authority public health teams; Infection Prevention and Control Nurses; and PHE regional health protection staff. These cuts to infrastructure and staffing pre-pandemic meant that staffing levels did not match the demand that health protection required.

Clive explained that pre-pandemic he had the impression that the EHO team linked to LA1 were managing, even though jobs had been cut. He had to acknowledge, however, during the pandemic, that this was not the case:

“… pre-COVID they [EHOs] seemed to be coping quite well, but it was only with COVID that you could appreciate that they were on the verge of being overwhelmed really.” (Clive, LAPHT, LA1)

In LA3, the arrival of COVID-19 drew attention to systemic gaps in health protection with respect to care homes. Pre-pandemic, LA3 did not have a dedicated Infection Prevention and Control Team for managing care home outbreaks and this led to particularities in the management of COVID-19 outbreaks in care homes in this local authority, as Philippa described:

“…And then the pandemic hit […] and I think what it did unearth that can of worms of that there was nobody really in charge for the care homes and that IPC [Infection Prevention and Control] element was hugely missing.” (Philippa, IPCT, LA3)

The arrival of the pandemic was seen by many participants to have unmasked weakened capabilities across all teams involved in local health protection, as Amy clearly articulated:

“…And I think what we have seen is basically a system that is struggling, […] and COVID has really shone a light on that because all of a sudden you had a system which was already overstretched then becoming under much more pressure both from the EHOs point of view and from the Infection Control Nurses and from the Public Health England point of view, to suddenly respond to this massive threat in terms of COVID.” (Amy, PHE/UKHSA, LA2)

#### Money could not ‘magic up’ expertise

The second way in which participants described COVID-19 had drawn attention to systemic issues in local health protection systems was by making visible the gaps in its workforce. When the extent and nature of the pandemic in England became apparent, there was a sudden injection of additional government funding into the COVID-19 response to quickly increase capacity in health protection work. Unfortunately, as Amy elucidated, health protection work is highly skilled, and requires intensive training. This meant it was difficult to quickly reverse the losses in health protection expertise in the preceding years:

“…. we have all seen there has been an injection of cash into ‘pandemic response’, … but what that doesn’t do is actually give you, it doesn’t magic up trained staff from places, you know you cut a budget you lose that staff and you lose that expertise and that experience from the system. And when you then need it back for things like COVID, it is not there…” (Amy, PHE/UKHSA, LA2)

Additional funding did not, therefore, lead to rapid increases in staff with health protection expertise to help with the pandemic response, because, as Helen made clear, they were very difficult to find:

“… it has not been easy to recruit to those roles. Probably after years of disinvestment both in public health teams and local authority environmental health public protection teams you know there is just not as many people around.” (Helen, LAPHT, LA1)

With no additional health protection expertise available, the extra money provided by the central government was most welcome, and this new investment was spent on bringing staff to strengthen the capacity of the health protection workforce. Thomas explained that they had no choice but to train these staff quickly, while simultaneously managing a COVID-19 response. This had a negative impact on both the quality of these staff’s training and on the workload of existing staff:

“…obviously you can’t pick Infection Control Nurses off a tree, you are talking two years before they are ready to run. So, we were bringing people in with this investment, and the whole programme of developing a new nurse just completely went out the window and we [were] taking chances. Cutting corners. And taking risks when we just normally wouldn’t do that.” (Thomas, IPCT, LA2)

#### Prioritising COVID-19 over other health protection activities

The third way that the pandemic exposed weaknesses in local health protection systems was by displacing routine health protection work. Participants gave multiple examples of how the pandemic led to routine work stopping completely, or being hugely reduced, including: partnership development work and reviewing standard operating protocols for non-COVID-19 diseases; inspecting food businesses; sampling in food businesses to check for pathogens; researching other infectious diseases; strategic work on Tuberculosis; infection prevention and control work in care homes for non-COVID-19 infections; work on antimicrobial resistance; and infection prevention training in nursing homes and hostels. Hannah described what these changing priorities looked like in practice:

“So yes, we certainly had a drop off the cliff really with regards to the work that we do, because it just wasn’t really there - everything was focussed on COVID …” (Hannah, PHE/UKHSA, LA3)

Sarah explained that during COVID-19 their routine programme of work, which involved inspecting food businesses to prevent food poisoning, had stopped for almost 12 months:

“…during COVID we didn’t do our routine programme [of inspecting food businesses] […] for the best part of a year we haven’t visited at all, we have only dealt with the absolute emergencies or failed tests, or in cases of food poisoning….” (Sarah, PPS, LA2)

This prioritisation of COVID-19 during this extended period, was, on the one hand, a deliberate, strategic decision. On the other hand, it was also due to staff having no capacity for anything else, as Rachel describes below:

“… to be realistic, it is very difficult to do anything other than COVID at the moment there is too much work to do.” (Rachel, PHE/UKHSA, LA2)

The one area of infectious disease work which had to continue during COVID-19 was reacting to outbreaks of non-COVID-19 infectious diseases. Perhaps due to social distancing rules, during COVID-19 the number of infectious diseases in the community did drop off [41]. There were still, however, several infectious disease outbreaks which had to be followed up. As Sally explains, this was exceedingly difficult and stressful for staff to manage alongside their COVID-19 work:

“… if you are in back-to-back meetings with COVID you haven’t got that capacity to be looking at other things, […] we had a legionnaires outbreak in the middle of all of this, […] it is juggling those resources and making sure you have got the skills and the expertise […] so, yes so it has been a struggle.” (Sally, PHE/UKHSA, LA3)

### COVID-19 as an opportunity

In the previous two themes, we illuminated the negative impacts of the pandemic on health protection systems in England. In our third and final theme, we go on to show how COVID-19 was also seen by participants as bringing positive change. First, because it increased the visibility of public health work, and infection prevention and control, second, because it had expanded infectious disease expertise in local authorities and third, because it increased local collaborations and strengthened working relationships.

#### Increasing the visibility of public health work and infection prevention and control

Many participants described how the pandemic had increased the prominence of work around infection prevention and control. Amy made clear that the pandemic meant that, for the first time, her parents understood what she did in her health protection role:

“I think we have got a higher profile than we had before. I think certainly my parents finally have figured out what I actually do for a job, (laughs) which has been reassuring.” (Amy, PHE/UKHSA, LA2)

As a wide range of public services across the country scrambled to understand what they needed to do to manage and mitigate COVID-19, health protection expertise was in high demand. The skills, knowledge and understanding of infectious diseases that consultants in public health, such as Alistair had, increased the credibility and relevance of his public health team and their services:

“… all of a sudden public health is very much at the top of everyone’s mind and I was the person being able to explain this is the incubation period of the virus, these are the symptoms, these are some of the control measures and then being able to translate that in really practical purposes […] I think that had a real impact on building the credibility of public health and people’s understanding of what public health could offer.” (Alistair, LAPHT, LA3)

As local health protection responders expanded their working networks to manage the pandemic, some participants became more aware of, and appreciative of the skills of other parts of the local health protection system. Clive explained that as COVID-19 had increased his work with Environmental Health Officers he had developed a greater understanding and appreciation of their skills:

“I think in terms of EHOs and public protection we have got a much greater appreciation of the scope of their work, and the breadth of the skills that they have got.” (Clive, PHT, LA1)

As a direct consequence of their increased profile, participants found themselves involved in more discussions around health protection work. Amy explained that she was asked to participate in advising re-commissioning Community Infection Prevention and Control, something she had not been asked to do pre-pandemic:

“… pre-pandemic I don’t know whether they [local authority partners] would have even necessarily have thought to involve the health protection team in those kinds of discussions, but because we are now on their radar we speak to them every day, several times a day, then actually we are able to provide a lot more influence and input into the system than we could before.” (Amy, PHE/UKHSA, LA2)

COVID-19 could therefore be seen to have raised awareness of infectious diseases, creating a greater appreciation of the work that health protection responders do, and providing opportunities for staff to integrate their expertise into a wider range of services.

#### Expanding Infectious disease expertise

The second way in which managing the pandemic was seen to have had a positive effect was through the expansion of infectious disease expertise.

Once the importance of the pandemic became apparent, health protection teams were given additional funding by the central government. As described earlier, this additional funding did not provide a ‘quick fix’ for the pandemic response with respect to staffing. Over time, however, this money did lead to recruitment of staff who, once trained, strengthened health protection capacity. For example, Clive explained that the pandemic had led to additional investment being put into Infection Prevention Control Services in his local authority:

“…on a positive note, I was just saying how the Infection Prevention Control Service was overwhelmed for a time, that did, stimulate additional investment, and a restructuring of the team so it is in much better shape now than it was 15 months ago.” (Clive, LAPHT, LA1)

Similarly, in LA3, which did not have a community Infection Prevention and Control Team with responsibility for care homes before the pandemic, COVID-19 money was used to build this team:

“…. it might have taken a pandemic but the system recognised the huge gap there was [in community Infection Prevention and Control for care homes] and then did something about it. And utilised the funds that were available to get the people in that could actually, help.” (Philippa, IPCT, LA3)

There was a recognition, however, that the extra staff recruited during the pandemic would not necessarily lead to an increase in capacity and capability long-term. The COVID money from the central government was short-term funding, so the increases in capacity might not be sustained, as Sally explains:

“…. we had so much money thrown at us, that we I think we went up 150% of staffing but, they were temporary contracts and they are all due to come to an end in September […] But that means all those staff are going to go.” (Sally, PHE/UKHSA, LA3)

Alongside this increase in health protection staff during the pandemic, there was also an expansion of health protection expertise in existing health protection responders. Pre-pandemic, most of the infectious disease outbreak work was led by PHE, leading to a perceived loss of skills in this area in some local authority public health teams. Since COVID-19, this situation had reversed, leading to the development of existing skills, as Helen outlines below:

“So, I am not historically the health protection lead, within the department. The health protection function has reduced for years, but over the last 12 months obviously we have all had to take on a lot more health protection type work as part of the COVID response. So, my own skills and experience have had to evolve quite quickly as well.” (Helen, LAPHT, LA1)

This evolution of skills in managing infectious diseases was echoed across several participants. Rachel described how, before COVID-19, outbreaks were managed by a small number of people, but COVID-19 meant that far more people, across several different teams, had increased their skills in this area:

“I think health protection was almost a niche skill or area of expertise but now it is greatly distributed. Which is good.” (Rachel, LAPHT, LA2)

At the height of the COVID-19 pandemic, therefore, there was an expansion of local health protection expertise, particularly within local authorities, facilitated by both increased staffing, and a wider distribution of infectious disease skills.

#### Increasing collaborations and strengthening relationships

The third silver lining of the pandemic was that it was seen to have increased collaboration and strengthened relationships within and beyond local health protection systems.

Participants felt that COVID-19 had improved collaborative working because, “*all hands-on deck*” (Hannah, LA3) were needed to support the pandemic response. Regular COVID-19 meetings, whether for management of outbreaks, or general COVID-19 oversight, increased and improved working relationships between PHE/UKHSA, local authority public health teams, Infection Prevention and Control Teams and Public Protection Services. For example, Alistair, a public health consultant working in the local authority in LA3 describes how COVID-19 work had changed the way he worked with EHOs:

“… I have seen far more of my Environmental Health colleagues over the last 2 years than I would have done beforehand just because of the need to organise to do prevention, the need to make sure that we are all consistent on the advice that we are giving, to invite them into the incident management team meetings and then them being able to do the more practical visits to settings to support them to get the right control measures in place.” (Alistair, LAPHT, LA3)

Miranda explained that regular online COVID-19 meetings had strengthened the links of the local authority public health team with PHE, which, before the pandemic had been limited to an occasional phone call:

“[before the pandemic] ... we had the telephone numbers to ring if we needed to talk to anybody or we had any issues so those links were there, they just weren’t as strong as they are now. […] So, that is a massive improvement...” (Miranda, LAPHT, LA3)

This increased collaborative working was something that participants thought had helped when managing COVID-19 and should be preserved going forward. LA3 had already been able to draw on the collaborations they had built during COVID-19 to respond to a recent avian influenza outbreak, as Andrew explained:

“… so, we did really well with avian flu because actually all the work we did within the COVID response so we actually had already built up really strong partnerships now with different organisations which they could help us with.” (Andrew, PPS, LA3)

The pandemic was also recognised to have improved working relationships outside the health protection system. Tracy described how the new relationships she had made and the existing relationships that she had cemented during COVID-19 would be invaluable to her work going forward:

“… I have to say we have got a lot of friends now, […] Internally there is not a bit of any of the bit of the council that we haven’t had to work with you know whether it is births and deaths registrations, or recycling colleagues and managing their outbreaks, libraries, so what we have done is expanded our network of partners […] and I am really grateful if there is anything positive that has come out of the pandemic for public health it has been that reach into all of the, all of the council, […] and I think that then does help you with other agendas or other diseases […] so it has changed how we work and will change how we work forever….” (Tracy, LAPHT, LA3)

## Discussion

In our introduction, we outlined Kruk and colleague’s (2015) definition of resilience in health systems as being *‘the capacity of health actors, institutions and populations to prepare for and effectively respond to crises; maintain core functions when a crisis hits; and, informed by lessons learned during the crisis, re-organise if lessons require it’* [6]. Below we apply each aspect of this definition of resilience to health protection, and examine each of these in relation to our findings.

### The capacity of health *protection* actors, institutions, and populations to prepare for and effectively respond to crises

UK pandemic preparedness measures date back to 1997 [8, 42, 43]. Despite this preparation, there was insufficient capacity within the local health protection teams included in our study to manage COVID-19 outbreaks, or the additional ‘COVID+’ work associated with the pandemic response.

Participants described how the loss of staff and expertise within local health protection systems in the years leading up to the pandemic hampered the effectiveness of the COVID-19 response. These responses were further hindered when it was not possible to re-instate the additional staff and expertise required imminently, or in a short timescale. This led to some tasks required to manage COVID-19 being passed to other teams with no specialist health protection expertise, potentially compromising the effectiveness of the local pandemic response.

The capacity of our participants to respond to COVID-19 was also hindered by the rapidly changing legislation and how this legislation was rolled out – centrally, with no prior communication to local health protection teams. The decision to introduce new national ‘COVID-19’ legislation rather than the use of the existing Civil Contingencies Act 2004 (CCA 2004) designed for the purpose of emergency responses has been critiqued by Mooavian (2021) and others and provided as evidence of governmental ‘panic’ [44]. One of the practical applications of COVID-19 regulations being disseminated from a central government source was that it hugely increased the workload of our participants as they had to constantly adapt to new guidelines, at very short notice, as they were released. Unsurprisingly, in these conditions, health protection responders were left feeling overwhelmed, and unable to cope, bringing their experience in line with frontline healthcare staff who also reported elevated levels of stress during the pandemic [11].

We argue that the ability of the local health protection systems in our study to respond to COVID-19, was undermined by structural conditions created by budget cuts imposed by central government in the years leading up to the pandemic. The systematic disinvestment in local health protection systems due to government austerity measures created a more fragile local health protection system which was less able to cope with the crisis [45]. This finding is consistent with a study from the United States of America which reported an underfunded and understaffed health protection system affecting their COVID-19 response [18]. Our findings may also be relevant to other comparable European countries such as Greece, Spain, Portugal and Ireland where austerity policies pre-pandemic have also led to significant reductions in health spending [46–48].

### Maintain core functions when a crisis hits

Our findings demonstrated that core health protection functions in the local authorities included in our study were significantly compromised during the COVID-19 pandemic. In prioritising COVID-19 work, almost all other work around infectious diseases ceased. The implications of this on the long-term health of local communities affected are not yet known, but it is likely that it may have a detrimental effect on infectious diseases unless additional measures are taken to compensate for the pause. One study looking at the impact of stopping TB services in three high-burden countries during the pandemic has estimated that unless catch-up mitigations are put in place, there will be millions more TB cases and hundreds of thousands more deaths [49].

### Informed by lessons learned during the crisis, re-organise if lessons require it

Re-organisations of health protection systems in the UK during the pandemic took place at both a central and local level. Centrally, the UK government: first, created lighthouse laboratories for COVID-19 testing; second, created a new national Test and Trace system for tracing contacts of COVID-19 cases; third, created the Joint Biosecurity Centre [50–52]; and fourth, merged PHE with Test and Trace and the Joint Biosecurity Centre – to create the UK Health Security Agency (UKHSA) [29–31]. An assessment of these central re-organisations is beyond the scope of this paper. The national test and trace system which cost £37 billion and was created from private companies rather than from existing expertise within health protection, was not, however, as successful as was hoped. A report by the House of Commons Committee of Public Accounts in March 2021 found that the NHS Test and Trace system, ‘had failed to deliver discernible benefits to the UK’s pandemic response’ [53]*.*

Despite central government re-organisations described above, our study gives examples of how locally, health protection systems were able to make positive changes to the management of infectious diseases. Infectious disease expertise and knowledge were expanded as a substantial amount of COVID-19 outbreak tasks were moved out of PHE and into local authority public health teams. This, in turn, led to increased collaborations and a strengthening of relationships which were described as being used to build more effective health protection responses going forward. The strengthening of relationships with local partners to manage COVID-19 suggests that future pandemic preparedness should also include decision-makers and leaders in local communities.

Prior to the pandemic, fragmentation of health protection responders across local authority public health teams, local authority Public Protection Services, Infection Prevention and Control Teams and regional PHE teams, through several re-organisations of these services, may have had an impact on collaborative working. Our finding that COVID-19 improved collaborations suggests that they were not working as effectively as they might have, before the pandemic. It is important that, going forward, these existing collaborations are not lost in further re-organisations of these services and that formal structures are put in place to preserve these collaborations if staff move on to different roles.

### Strengths and limitations

There are limitations to our study. As with all qualitative studies, its findings do not intend to be generalisable, but, instead, give an in-depth understanding of managing the pandemic from the perspective of health protection responders we interviewed. The timing of interviews, spanning several different waves of COVID-19 and various levels of restrictions and legislation creates diversity. Participants were describing their experiences of COVID-19 at distinct stages in the pandemic. This did, however, give us a variety of perspectives from staff actively involved in various pandemic responses. One ethical dilemma we had to navigate was the implications of taking health protection responders away from their critical pandemic work to take part in the study. To mitigate the impact of our research we were careful not to conduct interviews during peak waves of COVID-19 infection, and interviews were arranged online, to fit around participants’ work.

An important limitation in terms of the timing of interviews was that they took place while pandemic management was ongoing. The increased capacity in health protection that participants describe in our study is therefore specific to a certain time, rather than being indicative of long-term change. It is important that future research examines if any capacity built during the pandemic has been maintained.

Future research should also examine resilience in local health protection systems outside of a pandemic situation. Literature engaging with the concept of health system resilience increasingly suggests that it is those systems which are working effectively outside a crisis that are then the best at managing a crisis [4]. While it is a strength of our study that we interviewed participants while still dealing with the ‘crisis’ of the COVID-19 pandemic, research should also explore local health protection systems outside of crisis mode.

Although we recruited staff from a variety of contrasting local authorities, we did not recruit participants from rural local authorities, or all health protection professional groups (although main groups were included), potentially missing different perspectives. Future research should include the perspectives of all health protection professional groups and responders working in rural locations.

## Conclusion

With the appropriate resources and support, local health protection systems in England, with their skills and expertise in infectious disease prevention and control, could have been better prepared to manage the COVID-19 pandemic. However, budget cuts and the de-prioritisation of local health protection in the years leading up to the pandemic led to the local health protection responders in our study being left without the resources, capacity or infrastructure required to respond effectively to the COVID-19 pandemic. The ultimate test for a health protection system is how it responds to and manages future infectious disease threats, pandemics, and unexpected/unpredicted events/situations. Therefore, all health protection responders should have independence and be encouraged to have open, transparent and honest discussions so that lessons can be learnt from the current COVID-19 pandemic experience.

While our study may not be generalisable to all local authority health protection systems in England, due to the limited number of participants (*n*=20), our findings call into question the local system-wide preparedness for a public health emergency on this scale, and suggest a lack of resilience within local health protection systems. It seems crucial that, in preparation for future pandemics, scalable local health protection workforces are developed. This might include: an assessment of the existing skills mixes across teams and organisations; identifying training needs so that responses can be scaled up in public health emergencies; planning roles in advance for all stages of an emergency; and business contingency plans for core work to continue as staff are shifted onto emergency responses.

Pre-pandemic, it was estimated that funding needed to be increased by £1 billion per year to restore it to 2015/2016 levels [54]. Pursuing further austerity policies, as is currently planned in the UK [55] neither fills this gap nor builds pandemic preparedness, and may erode the small gains the COVID-19 pandemic has brought. Investing in public health infrastructure such as health protection, should happen even when we are not amid a pandemic [4, 45]. If the increased capacity built within local health protection systems in response to COVID-19 had been maintained through policy change this could have led to improved future pandemic preparedness. A report on public spending during the COVID-19 pandemic suggests, however, that most spending directly related to the COVID-19 pandemic ended after 2021/22 [56]. The increased capacity in local health protection described in this study, may not, therefore, have been maintained. Now the worst of COVID-19 is over, there are emerging signs that the ‘normalised crisis’ [45] of austerity will continue leading to a weaker health protection system preparedness. However, if health protection were reinstated as a core function and protected from government cuts, this would improve responsiveness and strengthen the health security of the nation. Health protection would then be better equipped to address emerging public health threats such as antimicrobial-resistant pathogens and those that could lead to future pandemics.

### Supplementary Information

**Additional file 1: S1.** GRIPP-2 Reporting.

**Additional file 2: S2.** Interview schedule.

## Data Availability

Data not available – participant consent Due to the potentially politically sensitive nature of the research, the participants of this study did not give written consent for their data to be shared publicly. Supporting data is not therefore available.

## Abbreviations

COVID-19: Coronavirus disease
EHO: Environmental Health Officer
HPA: Health Protection Agency
IPC: Infection Prevention and Control
IPCN: Infection Prevention and Control Nurse
IPCT: Infection Prevention and Control Team
PHE: Public Health England
LA: Local authority
LAPHT: Local authority public health team
NHS: National Health Service
OHID: Office for Health Improvement and Disparity
PPI: Patient and Public Involvement
PPS: Public Protection Services
SARS-CoV-2: Severe acute respiratory syndrome coronavirus 2
UK: United Kingdom
UKHSA: UK Health Security Agency
