# Supplementary material for: ‘A system that is struggling’: understanding health protection resilience in England during the COVID-19 pandemic through the experiences of local health protection responders
Source: BMC Health Serv Res. 2024 Feb 8;24:181. doi: 10.1186/s12913-024-10651-7 (PMC10854171; doi:10.1186/s12913-024-10651-7)
Supplement: Supplementary file 1 — Additional file 1: S1. GRIPP-2 Reporting. [file 12913_2024_10651_MOESM1_ESM.pdf]

S1: GRIPP2-Short Form

Section and Topic	Item	Page no.
**1. Aim**	The aim of Patient and Public Involvement (PPI) was to involve the public in all stages of the research project.	14
**2. Methods**	Ten members of the public were consulted across all stages of the project. These members of the public were recruited from the NIHR HPRU in GI infections PPI group http://hprugi.nihr.ac.uk/ and the ARC NW Coast PPI group https://arc-nwc.nihr.ac.uk/. Recruiting from both these groups meant that public contributors were recruited from diverse backgrounds. At the methods stage, PPI members were involved in:<br>• Refining the focus of the project.<br>• Advising on recruitment strategies.	13 & 14
**3. Study results**	The PPI team contributed to the study results in several ways including:<br>• Refining data analysis.<br>• Highlighting some of the limitations of the study.<br>• Highlighting the importance of having formal systems in place to aid health protection collaborative work in the future.	16-34
**4. Discussion and Conclusions**	Patient and Public Involvement in this study was effective and influenced important aspects of the study (see sections 2&3).<br><br>It was beneficial to this study that public partners were actively involved in several other studies in the HPRU and ARC and therefore had experience in this type of work. SR has involved a number of these partners in other projects, which has helped the relationships between partners and the research team develop outside this particular study.<br><br>Involving the same partners from the beginning to the end of the study to the end allowed partners to contribute to several different aspects of the study. This was facilitated by funding for partners' time through the NIHR HPRU-GI.<br><br>There were some limitations. Partners were not actively involved in recruiting participants to the study or coding the qualitative data. In future studies, the research team will look to involve partners in these aspects of the research study.	13-34
**5. Reflections/Critical perspectives**	PPI was embedded into many aspects of this study but with further thought could have been embedded further. Future studies could integrate training for public contributors in recruitment and qualitative coding so that they could be involved in more aspects of the study.<br>Partners appreciated good communication and facilitation from the research team. Partners stressed the importance of, and their appreciation of, being informed about where and how their contributions had made a difference.	n/a
