# Supplementary material for: ‘A system that is struggling’: understanding health protection resilience in England during the COVID-19 pandemic through the experiences of local health protection responders
Source: BMC Health Serv Res. 2024 Feb 8;24:181. doi: 10.1186/s12913-024-10651-7 (PMC10854171; doi:10.1186/s12913-024-10651-7)
Supplement: Supplementary file 2 — Additional file 2: S2. Interview schedule. [file 12913_2024_10651_MOESM2_ESM.pdf]

Supplementary File 2: Interview schedule

1. Interview schedule for participants with a direct role in Health Protection

I am interested in finding out how your role has changed over time, and the way in which local authority health protection systems respond to infectious diseases might have changed over time. I will ask a number of questions around this subject. The interview can be stopped at any time if you do not wish to continue. There are no right or wrong answers, I just want to find out what you think and what your experiences and practices have been.

<u>Roles and Responsibilities</u>

Background to role

- Can you tell me about your role in the local authority?
- How long have you been working in this role?
- Have you had any previous roles in this local authority? Can you tell me about them?
- Can you tell me what your roles and responsibilities are?
- Can you describe what you might do as part of that role in a typical day?
- Can you tell me about other things your role might involve?

Specific role in prevention and management of infectious diseases

- Can you tell me what role you have in the **prevention** of infectious diseases?
- Can you tell me what role you have in the **management** of infectious diseases?
- Can you tell me what organisations you work with to fulfil these roles and responsibilities?

Specific role in the prevention and management of Gastrointestinal infections

- Can you tell me what role you have in the **prevention** of gastrointestinal infections?
- Can you tell me what role you have in the **management** of gastrointestinal infections?
- Can you tell me what organisations you work with to fulfil these roles and responsibilities?

<u>Impact of local budget cuts and local authority restructuring on local authority health protection</u>

- Can you describe to me what impact (if any) local budget cuts and local authority restructuring have had on Environmental health/Public health services (use whichever appropriate to role) in the local authority you work in?

Prompts ask for examples of changes in, e.g.

a. Services provided?
b. Collaboration with other organisations?
c. Staff numbers?

- Can you describe to me what impact (if any) local budget cuts and local authority restructuring have had on the management or prevention of *infectious diseases* by Environmental health/Public health services (use whichever appropriate to role)

Prompts ask for examples of changes in, e.g.

a. Services provided?
b. Collaboration with other organisations?
c. Staff numbers?

- Can you describe to me what impact (if any) local budget cuts and local authority restructuring have had on the management or prevention of *gastrointestinal infections* by Environmental health/Public health services (use whichever appropriate to role)

Prompts ask for examples of changes in, e.g.

a. Services provided?
b. Collaboration with other organisations?
c. Staff numbers?

<u>Impact of COVID-19 on local authority health protection</u>

- Can you describe to me any changes in your roles and responsibilities since the COVID-19 pandemic?

- If you personally haven't had changes in your roles and responsibilities can you describe to me what impact (if any) COVID-19 have had on local authority public health services/environmental health services (delete as appropriate) as a whole?

Prompts ask for examples of changes in, e.g.

a. Services provided?
b. Collaboration with other organisations?

- Can you describe to me what impact (if any) COVID-19 have had on the management or prevention of other infectious diseases in the community by public health services/environmental health (delete as appropriate)?

Prompts ask for examples of changes in, e.g.

a. Services provided?
b. Collaboration with other organisations?

<u>Ask about other organisations, people or policies relevant to their role</u>

- Are there any people or organisations that we have not yet discussed who you think might be relevant to your role?

<u>Bring the interview to a conclusion</u>

- Is there anything else we haven't discussed that might be important to understanding how austerity or COVID-19 might have shaped the way infectious diseases are managed by local authority public health or Environmental Health services?

2. Interview schedule for participants with an indirect role in Health Protection (e.g. Health Protection managerial responsibilities)

I am interested in finding out how your role has changed over time, and the way in which local authority health protection systems respond to infectious diseases might have changed over time. I will ask a number of questions around this subject. The interview can be stopped at any time if you do not wish to continue. There are no right or wrong answers, I just want to find out what you think and what your experiences and practices have been.

<u>Roles and Responsibilities</u>

Background to role

- Can you tell me about your role in the local authority?
- How long have you been working in this role?
- Have you had any previous roles in this local authority? Can you tell me about them?
- Can you tell me what your roles and responsibilities are?
- Can you describe what you might do as part of that role in a typical day?
- Can you tell me about other things your role might involve?

Relevance of role to health protection services

- Can you tell me how your role relates to public health services and environmental health services in your local authority?
- Can you tell me how this role has changed over time?

<u>Impact of local budget cuts and local authority restructuring on local authority health protection services</u>

- Can you tell me if or how the resources you have available for health protection services (public health and environmental health) might have changed over the course of your time in [name of local authority]?
- Can you tell me what has been behind the changes in these resources over time?
  Prompts ask for examples of changes in, e.g.
    - Local budgets?
    - Changes to local authority structure?
- How has this influenced what has been prioritised or deprioritised?
- How have those decisions been made?
- Can you give me an example of how budget cuts have shaped the prioritisation/de-prioritisation of a specific service within health protection and how those decisions around that services were made?

<u>Impact of local budget cuts and local authority restructuring on the prevention and management of infectious diseases such as Gastrointestinal infections</u>

- Can you tell me if or how the resources you have available for the prevention and management of infectious diseases (such as gastrointestinal infections) might have changed over the course of your time in [name of local authority]?

- Can you tell me what has been behind the changes in these resources over time?
  Prompts ask for examples of changes in, e.g.
    a. Local budgets?
    b. Changes to local authority structure?
- How has this influenced what has been prioritised or deprioritised?
- How have those decisions been made?
- Can you give me an example of how budget cuts have shaped the prioritisation/de-prioritisation of infectious diseases within health protection and how those decisions were made?

Problems and Programs for health protection services in the local authority

- Can you tell me about the major problems that you are most occupied with which are relevant to health protection at the moment?
- Can you tell me about the major programs that you are most occupied with which are relevant to health protection at the moment?

Impact of COVID-19 on local authority health protection

- Can you describe to me any changes in your roles and responsibilities since the COVID-19 pandemic?
- Can you describe to me what impact (if any) COVID-19 have had on local authority public health or environmental health services as a whole?
  Prompts ask for examples of changes in, e.g.
    a. Services provided?
    b. Collaboration with other organisations?
- How has COVID-19 influenced what has been prioritised or deprioritised?
- How have those decisions been made?

Ask about other organisations, people or policies relevant to their role

- Are there any people or organisations that we have not yet discussed who you think might be relevant to your role?

Bring the interview to a conclusion

- Is there anything else we haven't discussed that might be important to understanding how budget cuts or COVID-19 might have shaped the way infectious diseases are managed by health protection services?

Following the Interview

Thank the interviewee for taking part in the interview and ask if they are happy to have his/her interview included in the research. Debriefing forms will then be given out.
